# Resensitizing the Untreatable: Zidovudine and Polymyxin Combinations to Combat Pan-Drug-Resistant *Klebsiella pneumoniae*

**DOI:** 10.3390/ph18101531

**Published:** 2025-10-11

**Authors:** Jan Naseer Kaur, Jack F. Klem, Gebremedhin S. Hailu, Nader N. Nasief, Yang Liu, Allison Hanna, Albert Chen, Patricia Holden, Shivali Kapoor, Nicholas M. Smith, Mark Sutton, Jian Li, Brian T. Tsuji

**Affiliations:** 1 UB Center of Infectious Diseases, University at Buffalo, Buffalo, NY 14214, USA; jfklem@buffalo.edu (J.F.K.); gebremed@buffalo.edu (G.S.H.); nnn2@buffalo.edu (N.N.N.); yliu343@buffalo.edu (Y.L.); achen273@buffalo.edu (A.C.); pnholden@buffalo.edu (P.H.); shivalik@buffalo.edu (S.K.); nmsmith2@buffalo.edu (N.M.S.); 2 School of Pharmacy and Pharmaceutical Sciences, University at Buffalo, Buffalo, NY 14214, USA; 3 Jacobs School of Medicine & Biomedical Sciences, University at Buffalo, Buffalo, NY 14214, USA; aehanna@buffalo.edu (A.H.); mdsutton@buffalo.edu (M.S.); 4 Monash Biomedicine Discovery Institute, Department of Microbiology, Monash University, Clayton, VIC 3800, Australia; jian.li@monash.edu

**Keywords:** *Klebsiella pneumoniae*, antimicrobial resistance, combination therapy, drug repurposing

## Abstract

**Background:** The emergence of pan-drug-resistant (PDR) *Klebsiella pneumoniae* has compromised the efficacy of last-line agents, leaving few therapeutic options. Repurposing zidovudine, an FDA-approved thymidine analog with antibacterial activity, may enhance existing therapies, but pharmacodynamic data under clinically relevant conditions are scarce. This study addresses this gap using static and dynamic in vitro models. **Materials/methods:** A PDR strain of *Klebsiella pneumoniae* harboring *bla*_NDM-1_, *bla*_CMY-6_, *bla*_CTX-M-15_, *bla*_SHV-2_, and disrupted *mgrB* was used in this study. Minimum inhibitory concentrations (MICs) followed by static time-kills were performed to investigate the synergistic interplay between zidovudine and last-line antibiotics (ceftazidime/avibactam, polymyxin B). To simulate human pharmacokinetics, a hollow-fiber infection model (HFIM) was employed using steady-state concentrations of zidovudine (4 mg/L), polymyxin B (4 mg/L), and avibactam (22 mg/L). Structural and morphological effects on bacterial cells were examined via fluorescence microscopy following glutaraldehyde fixation. **Results:** In this study, the PDR *K. pneumoniae* showed a ~5-fold reduction in polymyxin MIC when combined with zidovudine (from >4 µg/mL to 0.25 µg/mL). Time-kill assays demonstrated ≥2.5 log_10_ CFU/mL bacterial reduction with zidovudine-based combinations, whereas monotherapies failed to inhibit bacterial growth. In the HFIM, the triple combination achieved rapid bactericidal activity (>3 log_10_ CFU/mL reduction within 4 h) and sustained killing (>5–6 log_10_ reduction maintained through 216 h), with bacterial counts remaining below 1 CFU/mL. In contrast, dual combinations initially reduced bacterial burden (1–3 log_10_ reduction) but failed to maintain suppression, with significant regrowth (>10^10^ CFU/mL) observed by 168 h. Microscopy corroborated these findings, revealing extensive cellular damage in the zidovudine-containing treatment arms. These HFIM results underscore the potential of zidovudine-based triple therapy in overcoming resistance to last-line antibiotics in *K. pneumoniae*. **Conclusions:** Our results provide promising unprecedented insight into novel zidovudine-based combination therapies against difficult-to-treat MBL Gram-negatives. The observed synergy in MIC reduction, rapid killing in time-kill assays, and near-complete eradication in the HFIM underscore the therapeutic potential of this triple combination. Future studies will focus on broadening the application of these novel combinations to other ‘superbugs’, such as highly resistant strains of *Acinetobacter baumannii* and *Pseudomonas aeruginosa*.

## 1. Introduction

The global rise in antimicrobial resistance represents one of the most pressing challenges in modern medicine, with carbapenem-resistant Enterobacterales (CRE) emerging as a critical threat to public health [1,2]. Among these pathogens, *Klebsiella pneumoniae* has garnered particular attention due to its remarkable capacity to acquire and disseminate resistance mechanisms, leading to the emergence of pan-drug-resistant (PDR) strains that render most available antibiotics ineffective [1,3,4]. The therapeutic landscape for treating *K. pneumoniae* infections has become increasingly constrained. Traditional β-lactam antibiotics have been compromised by the widespread dissemination of extended-spectrum β-lactamases (ESBLs) and carbapenemases, including metallo-β-lactamases (MBLs) such as NDM-1. The introduction of newer β-lactam/β-lactamase inhibitor combinations, including ceftazidime/avibactam, offered initial promise but has been rapidly undermined by emerging resistance mechanisms [5]. Concurrently, polymyxins, once considered drugs of last resort, have faced increasing resistance through mechanisms, including *mcr-1* gene acquisition and *mgrB* gene disruptions, which modify lipopolysaccharide structure and reduce polymyxin binding [6,7,8,9,10].

The convergence of these resistance mechanisms in single isolates has created a perfect storm of pan-resistance, leaving clinicians with limited or no therapeutic options. This crisis has prompted researchers to explore alternative strategies, including drug repurposing and combination therapy approaches. Zidovudine, originally developed as an antiretroviral agent for HIV treatment, belongs to the class of nucleoside reverse transcriptase inhibitors. Beyond its antiviral properties, zidovudine has demonstrated unexpected antibacterial activity, particularly when used in combination with conventional antibiotics [11,12,13,14]. The mechanism underlying this antibacterial effect appears to involve interference with bacterial DNA synthesis and potential disruption of cellular processes that may enhance the efficacy of co-administered antibiotics. Previous investigations have suggested that zidovudine may potentiate the activity of polymyxins and β-lactam/β-lactamase inhibitor combinations against resistant Gram-negative bacteria [13]. However, these studies have been limited in scope, often lacking comprehensive pharmacodynamic evaluations under clinically relevant conditions. Furthermore, the specific activity against PDR *K. pneumoniae* strains harboring multiple resistance mechanisms has not been thoroughly characterized. The present study addresses these critical knowledge gaps by employing both static and dynamic in vitro models to comprehensively evaluate the synergistic potential of zidovudine in combination with last-line antibiotics against a PDR *K. pneumoniae* strain. Our investigation utilizes a clinically relevant isolate harboring multiple β-lactamases and colistin resistance mechanisms, providing insights that may directly translate to therapeutic applications.

## 2. Results

### 2.1. Antimicrobial Susceptibility and Synergy Testing

Initial susceptibility testing confirmed the pan-drug-resistant phenotype of the *K. pneumoniae* Nevada strain. The isolate demonstrated high-level resistance to polymyxin B (MIC > 4 µg/mL), ceftazidime (MIC > 32 µg/mL), and the ceftazidime/avibactam combination (MIC > 32/4 µg/mL), consistent with the presence of disrupted *mgrB* and multiple β-lactamase genes. Remarkably, the addition of zidovudine demonstrated significant synergistic activity with polymyxin B. When combined with zidovudine at sub-inhibitory concentrations, the polymyxin B MIC decreased from >4 µg/mL to 0.25 µg/mL, representing a greater than 16-fold reduction and indicating strong synergy (FICI = 0.125). This dramatic MIC reduction suggests that zidovudine effectively restores polymyxin sensitivity in this otherwise resistant strain. Similar synergistic effects were observed with other antibiotic combinations, though to varying degrees. The combination of zidovudine with ceftazidime/avibactam also demonstrated improved activity, though the effect was less pronounced than with polymyxin B combinations.

### 2.2. Static Time-Kill Analysis

**Figure 1:**
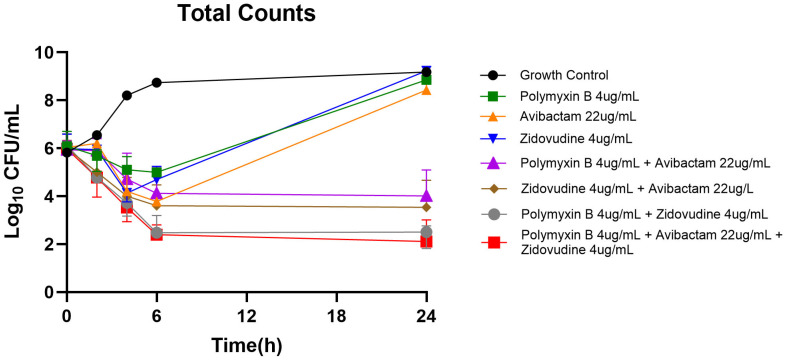
**Time-kill kinetics of zidovudine alone and in combination with avibactam and polymyxin B against *K. pneumoniae* Nevada.** Time-kill assays were conducted over a 24 h period to assess the antibacterial activity of zidovudine, avibactam, polymyxin B, and their combinations against *K. pneumoniae* Nevada. Bacterial counts (log_10_ CFU/mL) were measured at 0, 2, 4, 6, and 24 h. The limit of detection for the assay was 2 log_10_ CFU/mL.

Time-kill assays provided crucial insights into the kinetics of bacterial killing by individual agents and their combinations (Figure 1). Zidovudine monotherapy showed minimal bactericidal activity against the PDR strain, with bacterial counts remaining relatively stable throughout the 24 h observation period. Similarly, polymyxin B and avibactam monotherapies failed to achieve significant bacterial reduction, with counts remaining at or near starting levels. In striking contrast, combination therapies demonstrated substantial bactericidal activity. The combination of zidovudine with polymyxin B achieved >2.5 log_10_ CFU/mL reduction within 8 h, with continued bacterial suppression through 24 h. The zidovudine plus avibactam combination showed similar kinetics, achieving comparable bacterial reductions. Most remarkably, the triple combination of zidovudine, polymyxin B, and avibactam demonstrated superior inhibitory effect, achieving >3 log_10_ CFU/mL reduction within 4 h and maintaining sustained bactericidal activity through the remainder of the assay period.

### 2.3. Hollow-Fiber Infection Model Pharmacodynamics

**Figure 2:**
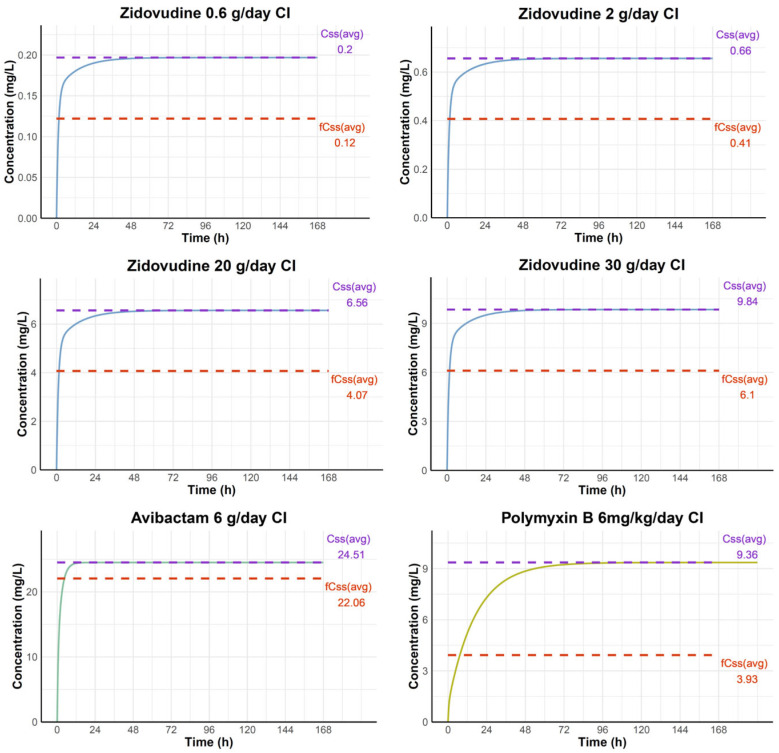
**Simulated concentration–time profiles during continuous infusion.** Pharmacokinetic simulations of zidovudine at 0.6 g/day, 2 g/day, 20 g/day, and 30 g/day, avibactam at 6 g/day, and polymyxin B at 6 mg/kg/day. Dashed lines indicate average steady-state total (C_ss_, purple) and free (*f*C_ss_, red) concentrations. Simulated *f*C_ss_ values were used to guide the HFIM experiments.

The HFIM studies provided critical validation of combination therapy efficacy under clinically relevant dynamic conditions. The simulated concentration–time profiles of zidovudine, avibactam, and polymyxin B under continuous infusion (CI) regimens are presented in Figure 2. PK simulations predicted that a CI of zidovudine at 0.6 g/day would result in an average steady-state total plasma concentration of 0.20 mg/L and a corresponding free concentration of 0.12 mg/L. At 2 g/day, zidovudine achieved total and free steady-state concentrations of 0.66 mg/L and 0.41 mg/L, respectively. At 20 g/day, total and free concentrations rose to 6.56 mg/L and 4.07 mg/L, while at 30 g/day, which was selected as the approximate upper limit of linear pharmacokinetics in humans [15], the predicted total and free concentrations increased to 9.84 mg/L and 6.1 mg/L, respectively. For avibactam at 6 g/day CI, the predicted total and free steady-state concentrations were 24.51 mg/L and 22.06 mg/L, respectively. At 6 mg/kg/day CI, polymyxin B reached total steady-state concentration of 9.36 mg/L and free concentration of 3.93 mg/L. PK profiling confirmed achievement of target steady-state concentrations for all agents throughout the treatment period. The free concentration of polymyxin B fell within the expert consensus target range of 2–4 mg/L considered acceptable from a toxicity standpoint [16]. Zidovudine concentrations remained stable at approximately 4 mg/L during the CI, representing clinically achievable free drug levels.

**Figure 3:**
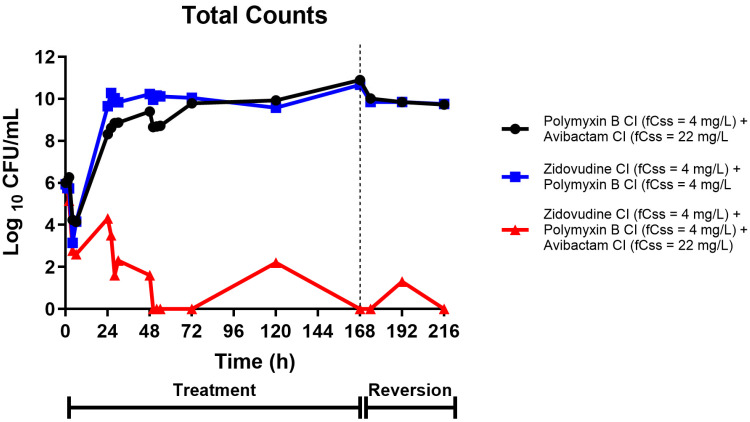
**Total bacterial counts during treatment and reversion phases in the HFIM.** Bacterial populations were monitored over 216 h in three treatment groups: Polymyxin B CI + Avibactam CI (black circles, targeted free steady state concentrations (*f*C_ss_) of 4 mg/L and 22 mg/L, respectively), Zidovudine CI + Polymyxin B CI (blue squares, both targeting an *f*C_ss_ of 4 mg/L), and Zidovudine CI + Polymyxin B CI + Avibactam CI (red triangles, targeted *f*C_ss_ values of 4 mg/L, 4 mg/L, and 22 mg/L, respectively). The treatment phase extended from 0 to 168 h, followed by a reversion phase from 168 to 216 h (indicated by timeline below x-axis). Bacterial counts are expressed as Log_10_ CFU/mL. The triple combination therapy (red triangles) demonstrated superior bactericidal activity with sustained suppression below detection limits, while dual therapies maintained bacterial counts around 10^10^ CFU/mL throughout most of the treatment period. CI = continuous infusion; *f*C_ss_ = free steady-state concentration.

The pharmacodynamic results revealed striking differences between treatment regimens (Figure 3). The dual combination of polymyxin B and avibactam (black circles) showed initial bacterial reduction of 1–2 log_10_ CFU/mL within the first 24 h, but this effect was not sustained. Bacterial regrowth occurred progressively, with counts exceeding 10^10^ CFU/mL by 168 h, indicating treatment failure despite continued antibiotic exposure. The combination of zidovudine with polymyxin B (blue squares) demonstrated improved initial activity compared to the polymyxin B/avibactam combination, achieving 2–3 log_10_ CFU/mL reduction in the first 48 h. However, this regimen also ultimately failed to maintain bacterial suppression, with substantial regrowth occurring after 72 h. In dramatic contrast, the triple combination of zidovudine, polymyxin B, and avibactam (red triangles) achieved rapid and sustained bacterial eradication. Within 4 h of treatment initiation, bacterial counts dropped by >3 log_10_ CFU/mL, progressing to 5–6 log_10_ CFU/mL reduction by 24 h. Most importantly, this bactericidal effect was maintained throughout the entire 168 h treatment period, with bacterial counts remaining at or below the limit of detection. During the reversion phase (hours 168–216), when all antibiotics were discontinued, the triple combination group maintained bacterial suppression below detectable limits, indicating potential bacterial eradication.

### 2.4. Microscopic Analysis

**Figure 4:**
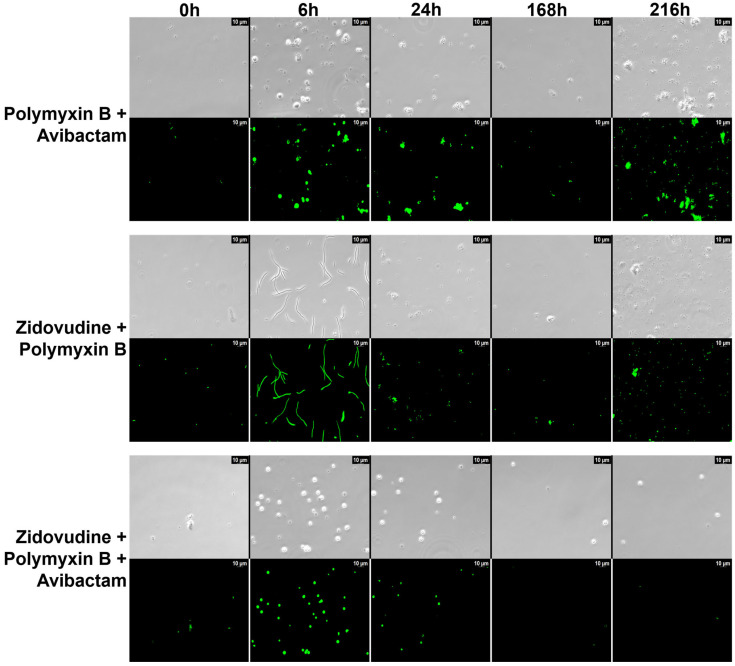
**Morphological changes in *K. pneumoniae* over time under different antibiotic treatments.** Representative images from the HFIM at multiple time points showing bacterial morphology by phase contrast (top row in each panel) and SYTO 9 fluorescence microscopy (bottom row in each panel). The zidovudine–polymyxin B combination induced prominent filamentation peaking at 6 h, followed by reversion to rod-shaped cells by 168 h. In contrast, the triple combination of zidovudine, polymyxin B, and avibactam resulted in rapid formation of spheroplast-like cells with no filamentation, and no morphological recovery observed through 168 h. Scale bar = 10 µm.

Morphological evaluation using SYTO 9 fluorescence microscopy revealed dynamic structural changes in response to antibiotic treatment that closely paralleled bacterial burden trends observed in the HFIM. In the zidovudine–polymyxin B combination, cells exhibited a pronounced filamentation phenotype by 6 h post-treatment, consistent with zidovudine’s inhibition of DNA replication (Figure 4). This filamentation peaked at 6 h, and by 24 h, very few viable cells remained, mirroring the rapid decline in CFU/mL in the HFIM. Morphological recovery began around 168 h, marked by a return to typical rod-shaped forms, coinciding with bacterial regrowth following drug reversion. In contrast, the triple combination of zidovudine, polymyxin B, and avibactam induced rapid conversion to small, rounded spheroplast-like forms, consistent with PBP2 inhibition by avibactam. Filamentation was absent, and importantly, no morphological reversion was observed even after drug washout. These spheroplasts remained structurally compromised, aligning with the sustained bacterial suppression seen in the HFIM through 216 h. These findings underscore the importance of avibactam in the triple combination; its inclusion not only introduces a distinct mechanism of morphological disruption but also prevents the regrowth and recovery seen with zidovudine and polymyxin B alone, leading to more complete and sustained bacterial killing.

## 3. Discussion

The results of this comprehensive investigation provide evidence for the therapeutic potential of zidovudine-based combination therapy against PDR *K. pneumoniae*. The dramatic synergistic effects observed across multiple experimental models underscore the promise of nucleoside analog repurposing as a strategy to combat antibiotic resistance [17]. To strengthen the link between our results and the central hypothesis, it is important to emphasize that multiple complementary experimental approaches consistently converged on the same outcome. MIC testing showed that zidovudine restored polymyxin susceptibility by >16-fold, directly supporting the hypothesis of resensitization. Time-kill assays demonstrated rapid bactericidal activity with zidovudine-based combinations, while all monotherapies failed, reinforcing the synergistic effect. Most critically, the hollow-fiber infection model validated these findings under dynamic, clinically relevant pharmacokinetic conditions, where the triple combination achieved sustained bacterial suppression through 216 h. Finally, microscopy provided morphological confirmation of irreversible structural damage in zidovudine-based regimens. Taken together, these lines of evidence strongly support our hypothesis that zidovudine potentiates the activity of last-line antibiotics against pan-drug-resistant *K. pneumoniae*. The mechanism underlying zidovudine’s antibacterial activity and synergistic interactions remains an active area of investigation. As a nucleoside analog, zidovudine may interfere with bacterial DNA synthesis pathways, potentially compromising DNA repair mechanisms that bacteria rely upon to survive antibiotic stress [18,19]. Additionally, zidovudine may alter bacterial cell wall permeability or interfere with efflux mechanisms, thereby enhancing the intracellular accumulation and activity of co-administered antibiotics.

The specific synergy observed with polymyxin B is particularly noteworthy given the disrupted *mgrB* mediated resistance of the test strain. The *mcr-1* gene encodes a phosphoethanolamine transferase that modifies lipid A, reducing polymyxin binding and conferring resistance [20,21]. The restoration of polymyxin sensitivity by zidovudine suggests potential interference with this resistance mechanism, possibly through direct inhibition of the Mcr-1 enzyme or indirect effects on lipopolysaccharide biosynthesis. The HFIM results provide crucial validation of therapeutic potential under clinically relevant conditions. The sustained bacterial eradication achieved by the triple combination, contrasted with the treatment failures observed with dual therapies, highlights the importance of multi-target approaches against highly resistant pathogens. The prevention of resistance development observed with the triple combination is particularly significant, as it suggests that this approach may not only treat existing infections but also prevent the emergence of even more resistant variants. From a clinical perspective, the concentrations used in this study are achievable with standard dosing regimens. Zidovudine has an established safety profile from its long-term use in HIV treatment, and the concentrations required for synergistic antibacterial activity are within the range achieved clinically. Polymyxin B and avibactam are both approved for serious Gram-negative infections, making this combination potentially translatable to clinical practice. The pharmacodynamic profiles observed in the HFIM studies suggest that this combination may be particularly valuable for treating serious infections such as pneumonia, bloodstream infections, and complicated urinary tract infections caused by PDR *K. pneumoniae*. The rapid bacterial killing observed would be advantageous in acute clinical scenarios, while the sustained suppression could prevent treatment failures and reduce the risk of recurrent infection.

Several limitations of this study should be acknowledged. The investigation focused on a single PDR *K. pneumoniae* strain, and broader testing against diverse resistant isolates will be necessary to establish the generalizability of these findings. Additionally, while the HFIM provides an excellent simulation of drug pharmacodynamics and pharmacokinetics, it cannot fully replicate the complexity of host immune responses and tissue-specific factors that influence therapeutic outcomes in clinical settings. Future research directions should include expanded testing against diverse carbapenem-resistant Enterobacterales, investigation of the molecular mechanisms underlying synergistic interactions, and evaluation of this combination in relevant animal infection models. The potential application to other problematic pathogens, including *Acinetobacter baumannii* and *Pseudomonas aeruginosa*, as mentioned in our conclusions, represents another promising avenue for investigation. The optimization of dosing regimens will also be crucial for clinical translation. While the current study employed continuous infusion-based regimens, clinical dosing involves complex pharmacokinetic profiling that may influence therapeutic outcomes. Pharmacokinetic-pharmacodynamic modeling studies will be essential to identify optimal dosing strategies that maximize efficacy while minimizing potential toxicity. In another research direction, polypharmacology approach, which relies on drugs that simultaneously act on multiple targets, offers additive or synergistic therapeutic effects while minimizing side effects. Indeed, dual-acting antibiotics have proven superior antibacterial efficacy and reduced vulnerability to bacterial resistance over single-target agents. Considering current data, the covalent ligation of zidovudine and polymyxin B represents an innovative strategy for next-generation antibiotic development, holding significant promise in overcoming the escalating threat of bacterial resistance [15,22,23].

The implications of this research extend beyond the specific combination studied. The successful repurposing of zidovudine for antibacterial applications demonstrates the potential of systematic drug repurposing approaches for combating antibiotic resistance. Many FDA-approved drugs possess untapped antimicrobial properties that could be leveraged through combination strategies.

**Limitations:** This study has important limitations. First, the findings are restricted to a single pan-drug-resistant *K. pneumoniae* isolate, and the generalizability to other resistant strains remains to be established. Second, the experiments were conducted exclusively In Vitro, using static time-kill assays and the hollow-fiber infection model. Although these approaches provide valuable pharmacodynamic insights, they cannot replicate the complexity of host immune responses, tissue penetration, or clinical pharmacokinetics. Third, while zidovudine concentrations used here fall within clinically achievable ranges, In Vivo efficacy, and optimal dosing regimens remain to be determined. Finally, resistance emerging beyond the experimental timeframe was not evaluated. Future studies in diverse clinical isolates, relevant animal infection models, and optimized dosing strategies will be essential to assess the translational potential of zidovudine-based triple therapy.

## 4. Materials and Methods

### 4.1. Minimum Inhibitory Concentration

MICs were determined using the broth microdilution method according to Clinical and Laboratory Standards Institute (CLSI) guidelines [16]. Two-fold serial dilutions of each antimicrobial agent were prepared in 96-well microtiter plates. Bacterial inoculum was prepared to achieve a final concentration of approximately 5 × 10^5^ CFU/mL in each well. Plates were incubated at 37 °C for 18–20 h, and MICs were defined as the lowest concentration that completely inhibited visible growth.

### 4.2. Static Time-Kill Assays

As previously described, time-kill studies were conducted over 24 h to evaluate the bactericidal activity of individual agents and their combinations [24]. Bacterial cultures were diluted to achieve starting inoculum of approximately 10^6^ CFU/mL in cation-adjusted Mueller-Hinton broth (CAMHB) containing test compounds at clinically relevant concentrations. Antibiotic solutions were added to achieve the desired drug concentrations (Polymyxin B, 4 mg/L; Avibactam, 22 mg/L; Zidovudine, 4 mg/L). Samples were incubated at 37 °C with constant agitation, and aliquots were removed at predetermined time points (0, 2, 4, 6, and 24 h) for viable count determination. Serial dilutions were plated on Mueller Hinton Agar plates, and CFU counts were determined after overnight incubation at 37 °C. Bactericidal activity was defined as ≥3 log_10_ CFU/mL reduction from the starting inoculum. Synergy was defined as ≥2 log_10_ CFU/mL reduction by the combination compared to the most active single agent.

### 4.3. Pharmacokinetics and Treatment Regimen Simulations

Suitable population pharmacokinetic (PopPK) models for zidovudine, avibactam, and polymyxin B were identified from the literature. For zidovudine, the two-compartment model described by Zhou et al. in phase II, multicenter, randomized, double-blind clinical trial was selected for its reliable design and large sample size [25]. Avibactam parameters were taken from the PopPK analysis by Li et al., which integrated data from multiple clinical trials and provided robust estimates based on a two-compartment model [26]. Polymyxin B parameters were obtained from the well-established two-compartment model reported by Sandri et al. [27]. Continuous infusion regimens were simulated over 7 days to predict plasma concentrations, including zidovudine at 0.6 g/day, 2 g/day, 20 g/day, and 30 g/day, avibactam at 6 g/day, and polymyxin B at 6 mg/kg/day. Free fractions of 62% [22], 90% [23], and 42% [27] were applied to calculate free steady-state concentrations (*f*C_ss_) for zidovudine, avibactam, and polymyxin B, respectively. All simulations were conducted in R (v4.4.2) using rxode2 (v3.0.4) for model execution, dplyr (v1.1.4) for data manipulation, and ggplot2 (v3.5.2) for visualization.

### 4.4. Hollow Fiber Infection Model

HFIM was employed to simulate clinically relevant pharmacokinetic profiles under dynamic conditions. *K. pneumoniae* Nevada was investigated in a 9-day HFIM using cellulosic cartridges (Cartridge C3008; FiberCell Systems Inc., Frederick, MD, USA), housed in a controlled incubation environment at 37 °C, as described previously [28,29,30,31,32]. The system was set up to simulate In Vitro a 2 h half-life with a total system volume of distribution of 135 mL. For the regimens tested, drugs were administered up to 168 h, after which the remaining drug in the system was allowed to be washed out with a 2 h half-life. Steady-state concentrations were achieved for polymyxin B (4 mg/L), avibactam (22 mg/L), and zidovudine (4 mg/L) through continuous infusion protocols. These concentrations were selected based on clinically achievable free drug concentrations in plasma and tissue compartments. The *K. pneumoniae* Nevada strain was inoculated to achieve starting bacterial densities of approximately 10^6^ CFU/mL. Treatment phases extended for 168 h, followed by a 48 h reversion phase where antibiotics were discontinued to assess the durability of treatment effects. Samples were collected at regular intervals throughout the experiment for bacterial enumeration and pharmacokinetic monitoring. Total bacterial counts were determined by plating serial dilutions on non-selective media, while resistant subpopulations were monitored using selective media containing relevant antibiotics.

### 4.5. Microscopic Analysis

Samples for imaging were collected from HFIM cartridges at baseline (0 h) and at multiple points post-treatment to assess morphological changes. Cells were fixed in 2.4% filtered glutaraldehyde (Electron Microscopy Sciences, Hatfield, PA, USA), gently mixed, and stored at 4 °C overnight prior to processing. For fluorescence imaging, 5 µL of the fixed sample was stained with 1 µL of SYTO 9 green nucleic acid stain and visualized using a Leica THUNDER microscope (Leica, Wetzlar, Germany) with a 20×/0.55 dry objective. Images were processed using Leica Application Suite X (LasX).

## 5. Conclusions

This study provides preclinical evidence that zidovudine-based triple combination therapy may represent a promising strategy against infections caused by pan-drug-resistant *K. pneumoniae*. Consistent signals across MIC reduction, time-kill assays, hollow-fiber pharmacodynamics, and microscopy underscore the potential of this approach to restore activity of last-line agents and achieve sustained bacterial suppression. However, these findings are limited to a single isolate and to in vitro systems. Broader evaluation in diverse resistant strains, relevant animal infection models, and optimized dosing studies will be critical next steps before clinical translation can be considered. These results therefore represent an important step toward assessing zidovudine-based triple therapy as a novel approach for tackling highly resistant Gram-negative pathogens, while highlighting the need for continued investigation to fully establish its therapeutic role.

## Data Availability

The original contributions presented in this study are included in the article. Further inquiries can be directed to the corresponding authors. The *Klebsiella pneumoniae* Nevada strain investigated in this study has been previously characterized by de Man et al. Findings from the genomic characterization of this isolate are available under BioProject accession number PRJNA391323 (https://www.ncbi.nlm.nih.gov/bioproject/PRJNA391323, first accessed on 10 January 2021). The chromosome, IncA/C2, IncFIB(pKPHS1), and IncFIB(K) genome sequences can be found under GenBank accession numbers CP022127, CP022126, CP022128, and CP022125, respectively. Further inquiries can be directed to the corresponding author(s).
